# Solution speciation and human serum protein binding of indium(III) complexes of 8-hydroxyquinoline, deferiprone and maltol

**DOI:** 10.1007/s00775-022-01935-6

**Published:** 2022-03-03

**Authors:** Orsolya Dömötör, Bernhard K. Keppler, Éva A. Enyedy

**Affiliations:** 1 Department of Inorganic and Analytical Chemistry, Interdisciplinary Excellence Centre, University of Szeged, Dóm tér 7, 6720 Szeged, Hungary; 2 MTA-SZTE Lendület Functional Metal Complexes Research Group, University of Szeged, Dóm tér 7, 6720 Szeged, Hungary; 3 Institute of Inorganic Chemistry and Research Cluster ‘Translational Cancer Therapy Research’, University of Vienna, Währinger Straße, 42, Vienna, Austria

**Keywords:** Stability constant, Albumin, Transferrin, Fluorescence, Ultrafiltration

## Abstract

Solution speciation and serum protein binding of selected In(III) complexes bearing *O,O* and *O,N* donor sets were studied to provide comparative data for In(III) and analogous Ga(III) complexes. Aqueous stability of the In(III) complexes of maltol, deferiprone, 8-hydroxyquinoline (HQ) and 8-hydroxyquinoline-5-sulfonate (HQS) was characterized by a combined pH-potentiometric and UV–visible spectrophotometric approach. Formation of *mono*, *bis* and *tris*-ligand complexes was observed. The *tris*-ligand complexes of HQ (InQ_3_) and deferiprone (InD_3_) are present in solution in *ca*. 90% at 10 µM concentration at pH = 7.4, while the *tris*-maltolato complex (InM_3_) displays insufficient stability under these conditions. Binding towards human serum albumin (HSA) and (apo)transferrin ((apo)Tf) of InQ_3_, InD_3_ and InM_3_ complexes and Ga(III) analogue of InQ_3_ (GaQ_3_) together with InCl_3_ was investigated by a panel of methods: steady-state and time-resolved spectrofluorometry, UV–visible spectrophotometry and membrane ultrafiltration. Moderate binding of InQ_3_ to HSA was found (log *K*′ = 5.0–5.1). InD_3_ binds to HSA to a much lower extent in comparison to InQ_3_. ApoTf is able to displace HQ, deferiprone and maltol effectively from their In(III) complexes. Protein binding of non-dissociated InQ_3_ was also observed at high complex-to-apoTf ratios. Studies conducted with the InQ_3_/GaQ_3_ – HSA – Tf ternary systems revealed the more pronounced Tf binding of In(III) via ligand release, while the original GaQ_3_ scaffold is preferably retained upon protein interactions and significant albumin binding occurs. Significant dissociation of InQ_3_ was detected in human blood serum as well.

**Graphical abstract:**

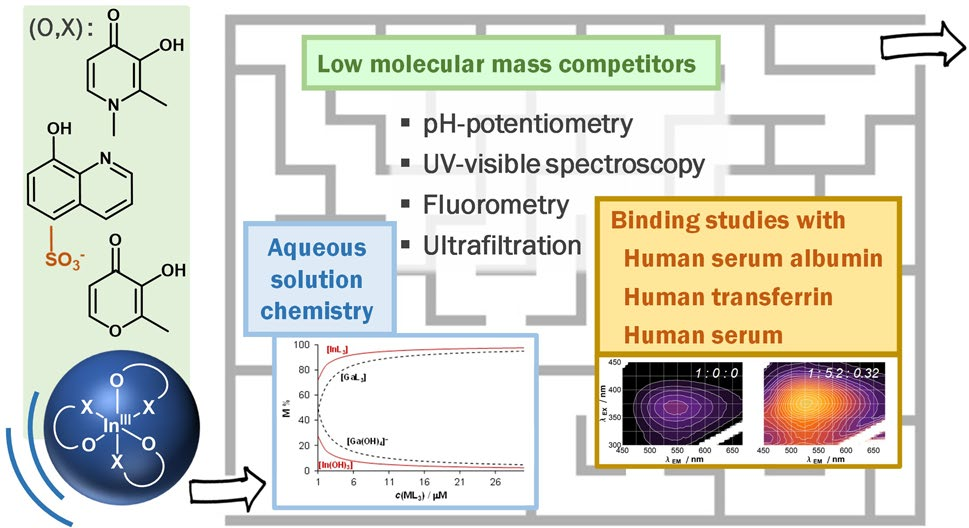

**Supplementary Information:**

The online version contains supplementary material available at 10.1007/s00775-022-01935-6.

## Introduction

Numerous In(III) and Ga(III) complexes are applied as pharmaceutical agents in connection with their diagnostic and therapeutic properties. Indium-based agents containing ^113m^In(III) or ^111^In(III) have been used in diagnostic nuclear medicine for decades [1–3]. A number of In(III)-chelates have been tested for the selective delivery of ^111^In(III) to various tissues, the *tris*-ligand complex of 8-hydroxyquinoline (HQ) is the only indium containing product currently approved by the FDA for the detection and diagnosis of infections and inflammatory lesions [2, 4]. This HQ complex is not directly injected in the blood, extensive serum protein binding of In(III) necessitates standardized procedures where cellular elements of the blood (principally leukocytes) are separated and labelled with the complex outside the patient’s body and then returned to the circulatory system [2, 5]. The HQ scaffold is found in various metal complexes (e.g. Cu(II), Fe(III) or ^89^Zr(IV)) being developed for cancer therapy or diagnostics [6, 7]. Recently anticancer activity of In(III) complexes of thiosemicarbazones and 3-hydroxy-2-methyl-4*H*-pyran-4-one (maltol) was reported on HepG2 (IC_50_ = 3.5 μM) and MDA-MB-231 (IC_50_ = 32 μM) cell lines, respectively [8, 9] and numerous In(III)-porphyrin complexes were tested for use in photodynamic therapy as well showing promising results [10, 11].

Radiopharmaceuticals containing ^67^ Ga and ^68^ Ga isotopes are used in imaging for some cancer types, infections and inflammatory diseases [12]. Besides, citrate stabilized salt Ga(NO_3_)_3_ (Ganite™) is a clinically approved formulation for the treatment of cancer-related hypercalcaemia [13]. *Tris*(8-hydroxyquinolinato)Ga(III) (GaQ_3_, often referred as KP46 in the literature) and *tris*(maltolato)Ga(III) (GaM_3_) are orally active antitumor metallodrugs recently undergone clinical trials [14, 15]. According to the hypothesized mechanism of action of GaM_3_, Tf-receptor mediated uptake of Ga(III) takes place, and it inhibits the ribonucleotide reductase enzyme [12]. In the case of GaQ_3_, Tf-receptor independent transport process is suggested and the complex activates a variety of mechanisms and pathways resulting in apoptotic cell death [16, 17].

Transferrin (Tf) is the most significant In(III) binding protein in blood serum and it is thought to compete effectively with chelate forming ligands like citrate, nitrilotriacetic acid (NTA), thioglycolic acid or HQ for the binding of In(III) ions [1, 18–21]. Ga(III) can bind to Tf as well, at the same time in vitro investigations conducted in our research group pointed out the impact of the chelate forming ligand on the competition between the ligand an this protein for Ga(III). Tf binding of Ga(III) predominates in the case of the less stable *tris*-maltolato complex and the original complex dissociates, while this process is less significant for the more stable GaQ_3_ [22]. Another difference is, that the In(III)-Tf adduct can bind to the Tf-receptors of the cell membrane, however, in contrast to Ga(III)-Tf, cannot be internalized by cells [23]. This form of In(III) is not available for cellular uptake but can reach possible use in monitoring of vascular permeability [20, 23, 24].

There are some studies dedicated to the Tf binding of In(III) complexes, while thermodynamic interpretations of the observations are scarcely provided, and interaction with other serum components (e.g. low molecular mass (LMM) constituents of blood or HSA) is rarely considered [18, 20, 23, 25, 26]. The Tf binding of In(III) ion was thoroughly investigated by Harris et al*.* and Kulprathipanja et al*.* via ligand competition studies, where the competitor ligand was NTA and EDTA, respectively [18, 26]. At the same time, the In(III)-Tf binding constants determined by the two research groups differ in more than 10 orders of magnitude. The possible albumin binding of In(III) complexes was not reported yet, although in the case of GaQ_3_ it was found, that the non-dissociated complex preferably binds to HSA under physiological conditions [22].

**Chart 1:**
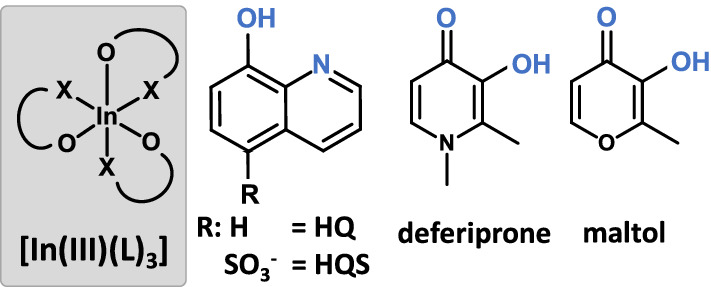
General structure of the *tris*-ligand In(III) complexes and chemical structure and abbreviations of the bidentate ligands 8-hydroxyquinoline (HQ), 8-hydroyquinoline-5-sulfonate (HQS), 3-hydroxy-1,2-dimethylpyridin-4(1*H*)-one (deferiprone) and 3-hydroxy-2-methyl-4*H*-pyran-4-one (maltol)

The available literature refers to somewhat different serum speciation of Ga(III) and In(III) complexes, which we found worth to investigate in more detail. In this work, our aim is to provide a detailed study on the serum speciation of a group of *tris*-ligand In(III) complexes, and compare these results to that of Ga(III) analogues. Therefore, we report the solution equilibria of In(III) complexes of HQ, 8-hydroxyqionoline-5-sulfonate (HQS), 3-hydroxy-1,2-dimethylpyridin-4(1*H*)-one (deferiprone) and maltol (see Chart 1 for the structures). Their stability is compared to that of the analogous Ga(III) complexes. Interaction of the *tris*-ligand complexes with LMM serum components such as citrate, oxalate and phosphate, and binding towards serum proteins Tf and HSA is investigated as well. Protein binding of the respective complexes was followed in binary and ternary systems and in blood serum by techniques such as steady-state and time-resolved fluorometry, UV–visible (UV–vis) spectrophotometry and membrane ultrafiltration–UV–vis to characterize the binding events and to provide a semi-quantitative description of the systems studied.

## Results and discussion

### Solution stability of In(III) complexes of HQ, HQS and maltol

**Fig. 1:**
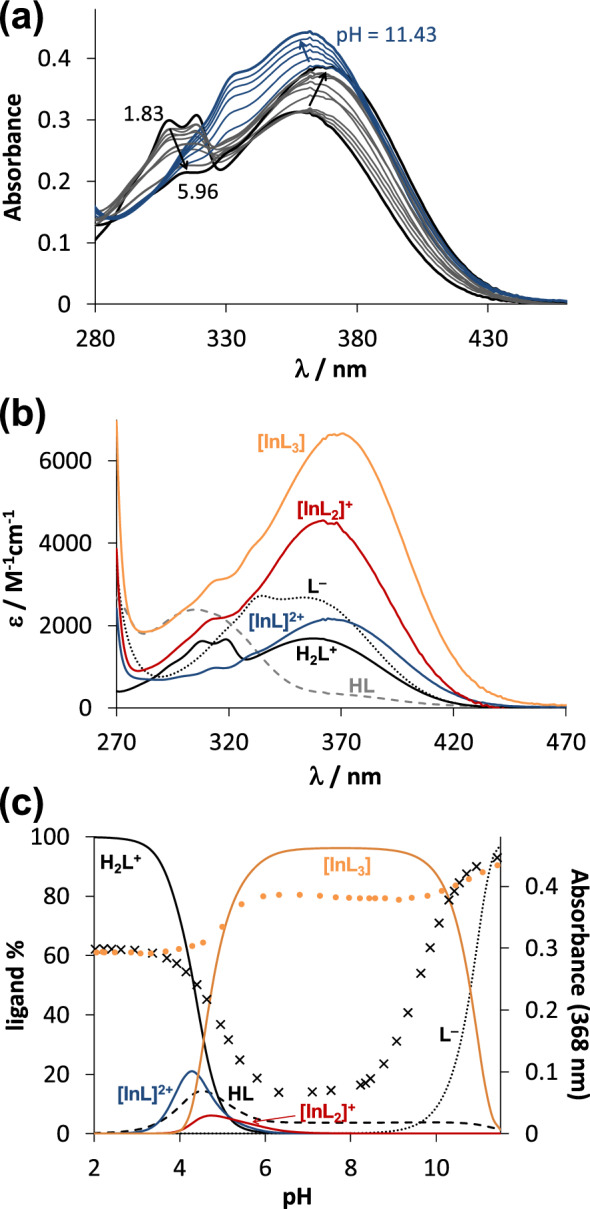
**a** UV–vis spectra recorded for the In(III) ‒ HQ (1:3) system at various pH values. **b** Computed molar spectra of the individual species. **c** Concentration distribution curves for the same system plotted together with the absorbance values at 368 nm (●) in addition to that of the metal-free ligand ( ×). {*c*_In(III)_ = 28.3 μM, *c*_L_ = 93.3 μM, *T* = 25.0 °C, *I* = 0.20 M (KCl), *ℓ* = 2 cm}

To characterize the solution stability of the In(III) complexes, pH-potentiometric and UV–vis spectrophotometric titrations were performed in water in the presence of 0.20 M chloride ions. However, stability constants for the HQ complexes could be obtained only by the latter method due to their much worse water solubility compared to the other compounds. HQS was involved as a water-soluble analogue of HQ bearing identical coordination mode. Stability constants of deferiprone were also determined by both methods in the present work to validate our experimental setup and data treatment, since the In(III) ‒ deferiprone system was already characterized by Orvig and collaborators [27]. The hydrolysis of In(III) and its interaction with chloride ions were taken into consideration, and stability constants of the hydoxido and chlorido complexes [28] were utilized for our calculations. As a first step, the proton dissociation constants (*K*_a_) were determined for the ligands (Table 1) showing good agreement with the previously published data (see Table 1) [29–31]. The overall stability constants obtained for the octahedral In(III)-deferiprone complexes by both methods (Table 1) are fairly similar to those reported at 150 mM NaCl [27]. As deferiprone, maltol, the other *O,O*-donor bearing ligand, also forms *mono*, *bis* and *tris*-ligand complexes; although they possess significantly lower log*β* values (Table 1) as it was observed for other metal ions such as Ga(III) [27, 29], Fe(III) [33, 34] or Zn(II) [31]. Complexes with the same stoichiometry were also formed with the *O,N*-donor containing HQS (Table 1). Notably, the determined overall stability constants obtained for the In(III) complexes of deferiprone, maltol and HQS by the two methods represent acceptable agreement. Stability constants for the HQ complexes (Table 1) were determined by the deconvolution of UV–vis spectra recorded at various pH values (Fig. 1a), and the computed molar absorbance spectra for the individual ligand and complex species are shown in Fig. 1b. Due to the lack of d-d or charge transfer bands, the molar absorbance spectra of the complexes represent similar λ_max_ values to that of the completely deprotonated form of the ligand; although, the shape of the spectra is different and is quite similar to that was found for the Ga(III) complexes [29].

**Table 1:** Logarithm of overall stability constants log*β* for In(III) complexes formed with deferiprone, maltol, HQ and HQS with the p*K*_a_ values of the ligands.^a^ (L is the completely deprotonated form of the ligands.) pM* (− log([M] + [M(OH)_(1–4)_]))^b^ were computed at pH 7.4 at 1 μM In(III) or Ga(III) and 10 μM ligand concentrations. (Charges of the various species are omitted for clarity.) {*T* = 25.0 °C, *I* = 0.20 M (KCl)}

	Method	Deferiprone	Maltol	HQS	HQ
p*K*_a_ (H_2_L)	pH-pot	3.66 ± 0.01^c^	‒	3.89 ± 0.02	4.99^d^
p*K*_a_ (HL)	pH-pot	9.72 ± 0.01^c^	8.47 ± 0.01	8.46 ± 0.02	9.51^d^
p*K*_a_ (H_2_L)	UV–vis	3.57 ± 0.02	‒	3.70 ± 0.04^f^	4.90 ± 0.02^g^
p*K*_a_ (HL)	UV–vis	9.70 ± 0.02	8.39 ± 0.02^e^	8.27 ± 0.04^f^	9.63 ± 0.02^g^
log*β* [InL]	pH-pot	13.67 ± 0.04^ h^	11.03 ± 0.03	12.64 ± 0.05	‒
log*β* [InL_2_]	pH-pot	23.50 ± 0.06^ h^	18.59 ± 0.03	21.31 ± 0.07	‒
log*β* [InL_3_]	pH-pot	32.66 ± 0.08^ h^	24.38 ± 0.05	28.95 ± 0.09	‒
log*β* [InL]	UV–vis	13.72 ± 0.03	10.77 ± 0.03	12.29 ± 0.06	13.43 ± 0.06
log*β* [InL_2_]	UV–vis	23.88 ± 0.06	18.78 ± 0.03	21.18 ± 0.06	22.87 ± 0.03
log*β* [InL_3_]	UV–vis	33.45 ± 0.03	25.04 ± 0.06	29.66 ± 0.06	32.64 ± 0.06
pIn*		7.95	6.01	7.93	8.18
pGa*		8.83^i^	6.00^j^	7.92^j^	7.86^j^

^a^Hydrolysis constants for In(III): log*β* [InH_‒1_] = ‒4.30, log*β* [InH_‒2_] = ‒9.40, log*β* [InH_‒3_] = ‒13.90, log*β* [InH_‒4_] = ‒23.40 [27]. Stability constants for In(III) chloride complexes: log*β* [InCl] = 2.32, log*β* [InCl_2_] = 3.62, log*β* [InCl_3_] = 4.00 [28]. ^b^Chlorido complex formation is negligible at these conditions. ^c^p*K*_a_ (H_2_L) = 3.67, p*K*_a_ (HL) = 9.77, *I* = 0.2 M (KCl) Ref. [31]. ^d^Taken from Ref. [29]. ^e^p*K*_a_ (HL) = 8.46, *I* = 0.2 M (KCl) Ref [29]. ^f^p*K*_a_ (H_2_L) = 3.63, p*K*_a_ (HL) = 8.48, *I* = 0.2 M (KNO_3_) Ref. [30]. ^g^p*K*_a_ (H_2_L) = 4.95, p*K*_a_ (HL) = 9.75, *I* = 0.2 M (KCl) Ref. [29]. ^h^log*β* [InL] = 13.60, log*β* [InL_2_] = 23.93, log*β* [InL_3_] = 32.93 at *I* = 0.15 M NaCl [27]. ^i^Computed based on stability constants taken from Ref. [27]. ^j^Computed on the basis of stability constants taken from Ref. [29], hydrolysis constants for Ga(III): log*β* [GaH_‒1_] = ‒2.46, log*β* [GaH_‒2_] = ‒5.92, log*β* [GaH_‒3_] = ‒10.63, log*β* [GaH_‒4_] = ‒16.87 [32]

**Fig. 2:**
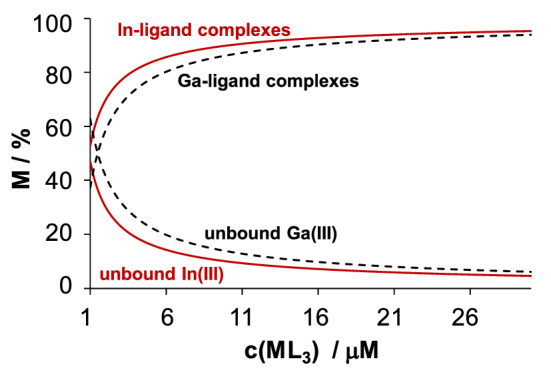
Estimated distribution of the *tris-*ligand HQ complexes of In(III) (solid lines) and Ga(III) (dashed lines) at pH 7.4 at various total concentrations. The majority (≥ 98%) of the ligand-bound complexes is present as InQ_3_ and GaQ_3_, and practically 100% of the unbound metal ions is found in hydroxido complexes. {*T* = 25.0 °C, *I* = 0.20 M (KCl)}

Based on the determined overall stability constants, concentration distribution curves were computed for the In(III) ‒ HQ system (Fig. 1c) representing the predominant formation of [InL_3_] complex (where L is the completely deprotonated form of the ligand) between pH 6 and 9.5. The same phenomenon was found for the analogous Ga(III) complexes [29]. Both metal ions form the *tris*-ligand complex at physiological pH, however, different isomers are present in solution, *mer*-isomers are predominant for Ga(III) while *fac*-complexes form with In(III) [35, 36]. The equilibrium concentration of the two *tris*-ligand complexes strongly depends on their total concentration as represented in Fig. 2. The fraction of the *tris*-ligand complex decreases somewhat more significantly with decreasing total concentration in the case of Ga(III). Despite the higher overall stability constants of Ga(III) complexes over the In(III) species, the stronger tendency of Ga(III) ions to hydrolysis results in a somewhat lower conditional stability at pH 7.4.

To compare the In(III) binding ability of the studied four ligands, pIn* (= negative decadic logarithm of the equilibrium concentration of the unbound metal species) values were calculated at pH 7.4 using the determined stability constants (Table 1). These values take into account the hydrolysis of the metal ions (Table 1, see Table S1 for pIn values) and can be compared to each other, which reveal the following stability trend for In(III): HQ > deferiprone, HQS >  > maltol. Similarly, pGa* values were also calculated (Table 1) representing a somewhat different stability order: deferiprone > HQS, HQ >  > maltol. The affinity of the studied ligands towards In(III) and Ga(III) do not differ considerably.

### Interaction of tris-ligand complexes with low molecular mass serum components

As a first step, interaction of the *tris*-ligand In(III) complexes with various LMM blood serum components was investigated and compared to that of GaQ_3_ studied formerly in our research group [22, 29]. The LMM compounds are considered to be more mobile than the high molecular mass components (HMM) such as proteins, and generally their reaction is characterized by faster kinetics. The role of serum compounds might have importance on the distribution of the In(III) complexes since they may be responsible for (partial) displacement of the originally coordinated ligand and they can also form mixed-ligand complexes. The complexes of In(III) and Ga(III) formed with HQ (InQ_3_, GaQ_3_), deferiprone (InD_3_) and maltol (InM_3_) were prepared in an aqueous solution by mixing the respective ligand and the metal ion in 3:1 ratio. The interaction of the In(III) complexes with serum components was followed at pH 7.40 in 10 mM HEPES containing 100 mM NaCl and 25 mM NaHCO_3_. All the measurements in this work were performed at 25 °C as most of the stability constants used for the modeling calculations published in the literature were determined at this temperature. UV–vis spectra recorded for the *tris*-ligand complexes at 10 μM concentration (see Fig. S1a for InQ_3_) in this buffer show 94%, 90% and < 20% non-dissociated InQ_3_, InD_3_ and InM_3_, respectively, which corresponds well to the fractions obtained using the stability constants determined at 0.20 M KCl: 90% (InQ_3_), 86% (InD_3_) and 9% InM_3_ and 6% InM^2+^. While InQ_3_ is somewhat more stable in comparison to GaQ_3_ (86%) [29]. InM_3_ is not stable enough at physiologically relevant low micromolar concentrations; therefore, no detailed investigations were conducted with this complex.

In(III) ion, as a hard Lewis acid, favors oxygen-donor chelating ligands, therefore, the effect of phosphate (1.1 mM), citrate (98 uM), and oxalate (9.3 uM), on the stability of InQ_3_ was investigated which are present in the blood plasma in considerable concentrations [37]. It was found that the UV–vis spectra of InQ_3_ were moderately affected by the addition of the mixture containing citrate, phosphate and oxalate in physiological concentration, and *ca.* 80% of the *tris*-ligand complex is present in its original form (Fig. S1), while the addition of tenfold excess of these LMM ligands resulted in significant dissociation of the complex (*ca*. 34% complex remained). In comparison, the Ga(III) complexes GaQ_3_ and GaM_3_ were practically not affected by the addition of the same LMM competitors in tenfold excess; although, the effect of the LMM components was investigated separately and not via the use of their mixture [22].

### Interaction of InQ_3_ and InD_3_ with human serum albumin

HSA is a universal transporter and the most abundant plasma protein with a concentration of *ca.* 630 μM in human blood plasma. It possesses three hydrophobic binding sites located in subdomains IIA, IIIA and IB, which are referred as the site I, II and III, respectively [38, 39]. There are metal-binding sites on HSA as well, *e.g*. multi-metal binding site, cysteine-34, and N-terminal site, however, these can provide coordination sites for metal ions of soft and borderline Lewis acid character (*e.g*. Hg(II), Pt(II), Cu(II), Zn(II)) [40, 41]. The binding of hard metal ions such as Al(III), Ga(III) and In(III) ions towards HSA are scarcely reported [20, 40]. This way, binding of the intact metal complexes on HSA via non-covalent bonds is more likely.

**Fig. 3:**
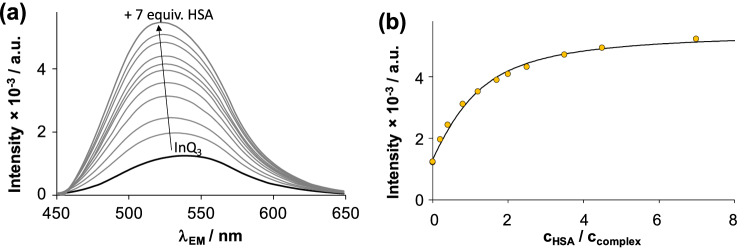
**a** Fluorescence spectra of InQ_3_ alone (thick black line) and in the presence of increasing amounts of HSA (grey lines). **b** Experimental (●) and fitted (solid line) intensities of the same system at λ_EM_ = 530 nm. {*c*_complex_ = 18 μM; *c*_HSA_ = 0 – 124 μM; *λ*_EX_ = 367 nm; pH = 7.40}

The effect of HSA on the intrinsic fluorescence of InQ_3_ and InD_3_ was studied. Similarly to GaQ_3_ and *tris*(8-hydroxy-quinolinato)Al(III) [22, 42], InQ_3_ and InD_3_ are also fluorescent metal complexes (see Table 2 for λ_max_ values). We have found that the fluorescence of InQ_3_ does not overlap with that of HSA, which makes the interpretation of the measured emission spectra (Fig. 3) easier. The fluorescence intensity of InQ_3_ increased in the presence of HSA, and emission intensity values measured at 530 nm show a saturation character plotted against the added HSA equivalents. The interaction was found to be rapid as it took place within a few seconds. At the same time, UV–vis absorbance spectra of InQ_3_ are only slightly altered by the binding interaction. Enhanced fluorescence of InQ_3_, and fast binding kinetics confirm the binding of the non-dissociated metal complex to the protein via intermolecular interactions. Fluorometric data can be excellently fitted with the computer program PSEQUAD [43] assuming a single site model (see Fig. 3), and a conditional binding constant of log *K*′ = 5.0 ± 0.1 was calculated for the HSA – InQ_3_ adduct. This constant indicates a moderate affinity of the complex on HSA. GaQ_3_ itself binds to HSA, but a considerably lower binding constant was determined for this complex (log*K*’ = 4.04), while GaM_3_ showed no interaction with this protein [22]. The fluorescence spectra of InD_3_ and HSA overlap significantly (see Fig. S2); although the intensities are additive, therefore, no considerable binding of InD_3_ on HSA is suggested. Ultrafiltration experiments revealed that only *ca.* 8% InD_3_ complex was bound to 2.5 equiv. protein and interestingly small deferiprone liberation refers to the binding of *ca.* 7% In(III) ion to the protein in addition [20].

**Table 2:** Excitation (λ_EX_) and emission maxima (λ_EM_) of InQ_3_ and GaQ_3_ complexes and fluorescence lifetime (*τ*_i_) and amplitude (*α*_i_) values alone and in the presence of HSA in various concentrations at pH = 7.40. {*c*_complex_ = 10 μM; *c*_HSA_ = 2–10 μM; *T* = 25.0 °C; in 10 mM HEPES, 25 mM NaHCO_3_, 100 mM NaCl buffer}^a^

	InQ_3_^b^	GaQ_3_
λ_EX max_ (nm)	254, 370	367^c^
λ_EM max_ (nm)	540	532 ^c^
Fluorescence lifetime parameters of the complexes alone		
* τ*_1_ (ns)	0.80 ± 0.03	0.54 ± 0.04
* τ*_2_ (ns)	1.41 ± 0.07	1.23 ± 0.03
* α*_1_ (%)	75 ± 4	57 ± 5
* α*_2_ (%)	25 ± 4	43 ± 5
Two additional lifetime components in the presence of HSA		
* τ*_3_ (ns)	5.7 ± 0.3	5.0 ± 0.3
* τ*_4_ (ns)	13.8 ± 0.6	14.0 ± 0.2

^a^Values are the average of at least three measurements. ^b^Data for InD_3_: *λ*_EX max_ = 295 nm, *λ*_EX max_ = 354 nm. ^c^Data taken form Refs. [22, 29]

Binding at the site I hydrophobic pocket of HSA can be followed via the selective excitation (λ_EX_ = 295 nm) of the single Trp (Trp-214) located close to this site. Trp-214 is sensitive to the binding events taking place at or near this site. The InQ_3_ complex quenched effectively the fluorescence of HSA, and a quenching constant (*K*’_Q_) could be computed based on the measured spectra: log *K*’_Q_ = 5.1 ± 0.1. This quenching constant is rather similar to the constant obtained from spectral data collected at λ_EX_ = 370 nm (vide supra), which strongly suggests the existence of a single binding site of InQ_3_ on HSA at the site I. The quenching of Trp-214 fluorescence most probably originates from (fluorescence) resonance energy transfer (FRET, or RET) mechanism, more details on this phenomenon can be found in Fig. S3 [44].

The fluorescence lifetime of InQ_3_ was determined in the absence and presence of HSA. InQ_3_ possesses a bi-exponential fluorescence decay characterized by *τ*_1_ = 0.79 ± 0.03 ns and *τ*_2_ = 1.39 ± 0.05 ns; and the amplitudes (α_*i*_) of the two components practically did not depend on the applied concentration of the complex in the 5–20 μM range. GaQ_3_ behaves similarly (Table 2), while lifetimes of InD_3_ were too short (*τ* ≤ 0.2 ns) for accurate determination with our instrument. By the addition of HSA to the complex, two additional lifetime components appeared for InQ_3_: *τ*_3_ = 5.7 ± 0.3 ns and *τ*_4_ = 13.8 ± 0.6 ns, which correspond to the HSA bound forms. The two ‘bound’ lifetimes do not consequently mean the presence of two distinct binding sites, but possibly reflects the bi-exponential decay of the complex [45]. The GaQ_3_ ‒ HSA system could be characterized with similar lifetime parameters (Table 2).

### Interaction of InQ_3_ and InD3 with human transferrin

Transferrin is the major iron transporter in blood and consists of two domains each possessing an iron-binding site called C- and N-terminal sites. Tf is not completely saturated with Fe(III) under physiological conditions; and approximately 70% of the binding sites are available for various metal ions. The binding of several metal ions to apo-transferrin (apoTf, iron-free Tf) has been investigated and trivalent metal ions, *e.g.* Al(III), Ga(III), In(III) or lanthanide ions, can bind with high affinity at the iron-binding sites [19, 23, 37, 46, 47]. Interaction of metal complexes with (apo)Tf, if it takes place, is most likely competition between the original ligand(s) and (apo)Tf for the metal ion.

Firstly, the kinetics of the reaction between apoTf and InCl_3_ and the complexes was studied by UV–vis spectrophotometry. The reaction of 2 equiv. InCl_3_ with apoTf took *ca*. 2 h, and the presence of excess citrate (10 equiv. to InCl_3_) did not considerably increase the reaction rate (Fig. S4). These observations and spectral changes displayed by apoTf in the presence of In(III) ions (Fig. S4) agree well with the findings of Battistuzzi et al*.* [25]. At the same time Harris et al*.* have observed slow equilibration with InCl_3_, that was accelerated (to 6 h) in the presence of NTA [18]. In the case of InQ_3_ and InD_3_, the progress of apoTf binding could be followed through the ligand bands of the complexes. The equilibrium was reached in *ca*. 3–4 h and 0.5 h for InQ_3_ and InD_3_, respectively. The Ga(III)-analogue GaQ_3_ interacted within 1 h with apoTf under similar conditions [22].

**Fig. 4:**
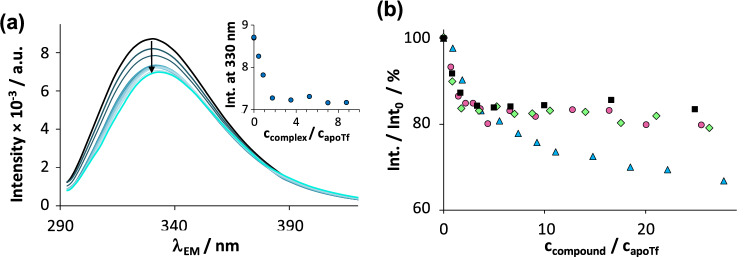
**a** Fluorescence emission spectra of apoTf in the presence of various amounts of InD_3_, inset shows the intensity changes at 330 nm. **b** Comparison of the fluorescence intensities of apoTf by addition of InCl_3_ (■), InM_3_ (●) InD_3_ (♦) and InQ_3_ (▲) at 330 nm plotted against the ratio *c*_compound_ / *c*_apoTf_. {*c*_apoTf_ = 1 μM, *c*_compound_ = 0 – *ca.* 30 μM; *λ*_EX_ = 280 nm; pH 7.40 equilibration time: 1 h (InD_3_), 3 h (InCl_3_, InM_3_), 6 h (InQ_3_)}

Fluorescence quenching is a convenient technique to study the binding of metal ions at the iron-binding sites of apoTf since it results in reduced fluorescence intensity of Tf. Batch samples containing 1 μM apoTf and various amounts of In(III) compounds were prepared. As Fig. 4a shows, the addition of InD_3_ quenched the fluorescence intensity of apoTf, the emission maximum was slightly red shifted and the emission stabilized at *ca.* 83% of the initial intensity. The quenching curve shown at *λ*_EM_ = 330 nm (inset of Fig. 4a) possesses a definite breakpoint at complex-to-Tf ratio of *ca.* 2, which suggests the competitiveness of Tf with deferiprone for the binding of In(III). Rather similar behaviour was observed for InCl_3_ and InM_3_, which later is almost completely dissociated at this concentration (Fig. 4b). The partial (and not complete) quenching of apoTf can be explained by the presence of numerous Tyr (26) and Trp (8) residues in the protein among which only a few are affected by the binding of In(III) at the iron-binding sites [48]. Upon addition of InQ_3_, the quenching curve is more stretched and aims to lower intensities (Fig. 4b). Interaction of the non-dissociated complex InQ_3_ with apoTf cannot be excluded, the latter proposed interaction is realized via intermolecular binding mode and more likely takes place on site(s) other than the iron-binding pockets (vide infra).

**Fig. 5:**
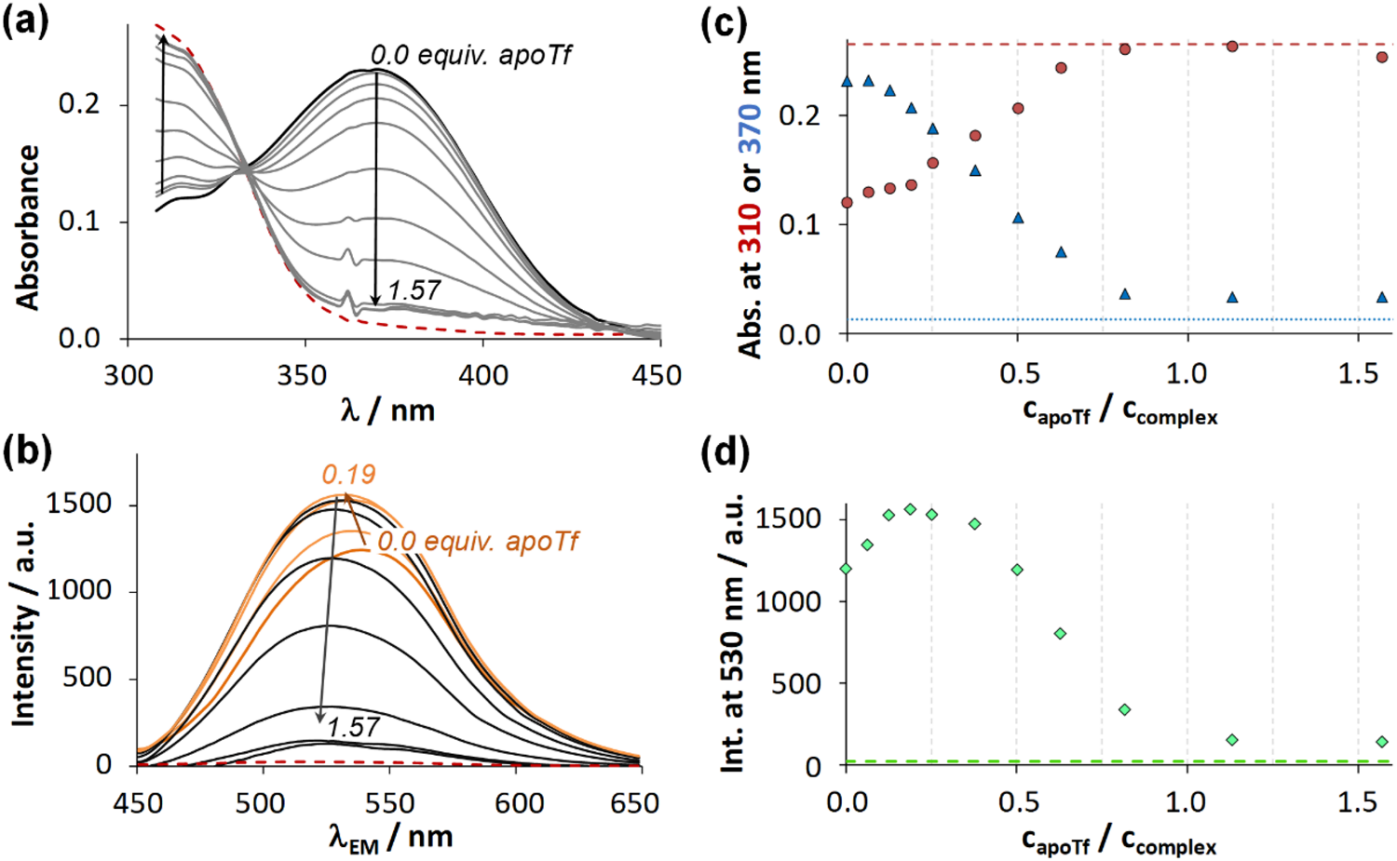
**a** UV–vis absorbance and **b** fluorescence emission spectra recorded for InQ_3_ in the absence and presence of various amounts of apoTf, the spectrum of free HQ (red dashed line) is plotted as well. **c** Changes of the absorbance at 310 nm (●) and 370 nm (▲) (red dashed and blue dotted lines denote the absorbance belonging to free HQ at 310 nm and 370 nm, respectively). **d** Fluorescence intensity at *λ*_EM_ = 530 nm of the same system (♦) (while the green dashed line shows the emission intensity of HQ). All the absorbance spectra are subtracted by the spectrum of apoTf. {*c*_complex_ = 19 μM; *c*_apoTf_ = 0 – 30 μM; *c*_HQ_ = 57 μM; *λ*_EX_ = 367 nm (**c**); *ℓ* = 2 cm (**a**, **c**), 1 × 1 cm (**b**, **d**); pH = 7.40, equilibration time: 6 h}

Complex InQ_3_ has a characteristic absorbance band at 370 nm and it is fluorescent as well, while ligand HQ absorbs light at shorter wavelengths (*λ*_max_ = 305 nm) at pH 7.4 and displays negligible fluorescence. Therefore, UV–vis and fluorescence emission spectra of InQ_3_ were recorded in the presence of increasing amounts of apoTf (Fig. 5a, b). After a short initial phase, the absorbance band of InQ_3_ is reduced gradually by the addition of the protein, while the development of the new band at lower wavelengths (at *ca.* 310 nm) refers to the liberation of HQ. Namely, there is a competition for In(III) ion between the ligand and the iron-binding sites of apoTf. Absorbance values depicted at 370 nm and 310 nm (Fig. 5c) saturate roughly to the corresponding values of the free ligand and outline a sigmoidal shape. Fluorescence intensity of the same sample set (Fig. 5b, d) was enhanced and moderately blue shifted (from *λ*_max_ = 540 nm to 532 nm) until the addition of *ca.* 0.2 equiv. apoTf. Similar tendency was observed for InQ_3_ when it was titrated by HSA (vide supra, Fig. 3). Then, by the addition of more apoTf, intensities decreased and *λ*_max_ shifted further to shorter wavelengths (527 nm). The interaction between apoTf and dissociated HQ can be excluded since no this kind of spectral changes could be observed for HQ – apoTf samples. Based on this and the results of apoTf quenching (vide supra) protein binding of the non-dissociated complex is presumed. This is further supported by fluorescence lifetime measurements; where new lifetime components τ_3_ = 4.7 ± 0.2 ns and τ_4_ = 13.8 ± 0.4 ns appeared for InQ_3_ in the presence of 0.1 – 0.3 equiv. apoTf (after 1 min waiting time). These values are fairly similar to those observed for the InQ_3_ – HSA system (*cf*. τ_3_ = 5.7 ns, τ_4_ = 13.8 ns). In all, binding of the intact complex to apoTf is apparent at high complex excess preventing partly the coordination of In(III) at the iron-binding sites, then the latter process becomes more pronounced at higher apoTf concentrations. Samples were prepared also with Tf (loaded with iron in *ca.* 20–40%). Here, the sigmoid shape of the UV–vis absorbance was seen again (Fig. S5) and the shifted inflection point in comparison to the titration with apoTf underlines the lower In(III) binding capacity of Tf. Interestingly, the fluorescence of these samples was practically unaltered till the addition of *ca.* 0.3 equiv. Tf. Binding of GaQ_3_ via intermolecular interactions to apoTf and Tf was also investigated for comparative reasons in this work, however, no such binding was observed.

**Fig. 6:**
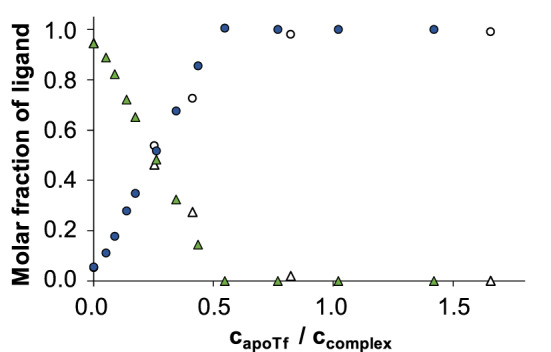
Molar fractions of complex bound (InD_3_, ▲,Δ) and free deferiprone (●,○) calculated on the basis of UV–vis spectra recorded for the InD_3_ – apoTf system (filled symbols) and results of the ultrafiltration–UV–vis experiments (empty symbols) are plotted as well. {*c*_complex_ = 22 μM (UV–vis), 19.8 μM (ultrafiltration); pH = 7.40}

In the case of InD_3_, the absorbance bands of the metal complex and apoTf overlap in great extent, however, spectra can be deconvoluted to the spectra of InD_3_, deferiprone and apoTf (see Fig S6 for details), and molar fractions of the respective species can be calculated (Fig. 6). Ultrafiltration experiments were carried out as well for the InD_3_ – apoTf system and the non-protein bound LMM fractions were analyzed by UV–vis spectrophotometry (Fig. 6). These ultrafiltration measurements corroborate the findings of the spectrophotometric assay. The complex dissociated in line with the addition of apoTf and a practically quantitative displacement of deferiprone seems to take place by the addition of *ca.* 0.5 equiv. apoTf. Namely, both of the iron-binding sites participate in the binding of the In(III) ion.

Noteworthy, the competitiveness of apoTf for In(III) with the ligands deferiprone and HQ seems to be more significant as it would be assumed on the basis of stability constants reported for the In(III) – Tf system by Harris et al. (log*K* (In-Tf) = 18.74 and log*K* (In_2_-Tf) = 16.86) [18]. Our results suggest about 2–3 orders of magnitude higher stability constants for the latter system under the conditions applied for the studies.

### Speciation of InQ_3_ and GaQ_3_ in the presence of both serum proteins and in serum

**Fig. 7:**
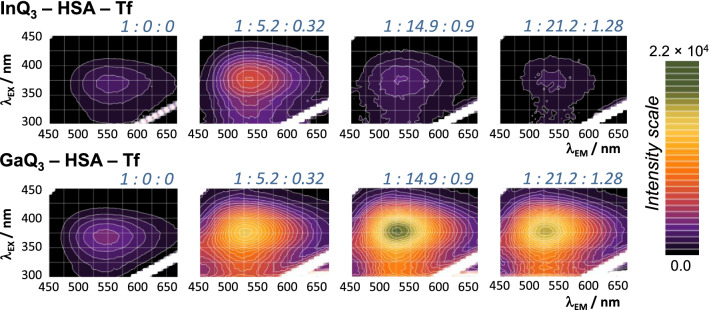
Three-dimensional fluorescence spectra recorded for InQ_3_ – HSA – Tf and GaQ_3_ – HSA – Tf ternary systems at the indicated compositions. Numbers indicate the complex-to-HSA-to-Tf ratios. The ratio of the two proteins corresponds to their physiological ratio in the blood serum. {*c*_InQ3_ = *c*_GaQ3_ = 10 μM, *c*_HSA_ = 0 – 212 μM; *c*_Tf_ = 0 – 12.8 μM; pH = 7.40, equilibration time: 6 h; spectra are corrected by self-absorbance and inner filter effect}

The reliability of UV–vis and fluorescence spectroscopic techniques in the characterization of the mode of interaction of In(III) complexes with HSA and (apo)Tf was proven in the former sections. Therefore, as a continuation three-dimensional fluorescence and UV–vis spectra were recorded for InQ_3_ and GaQ_3_ in the presence of both HSA and Tf (Figs. 7 and S7).

**Fig. 8:**
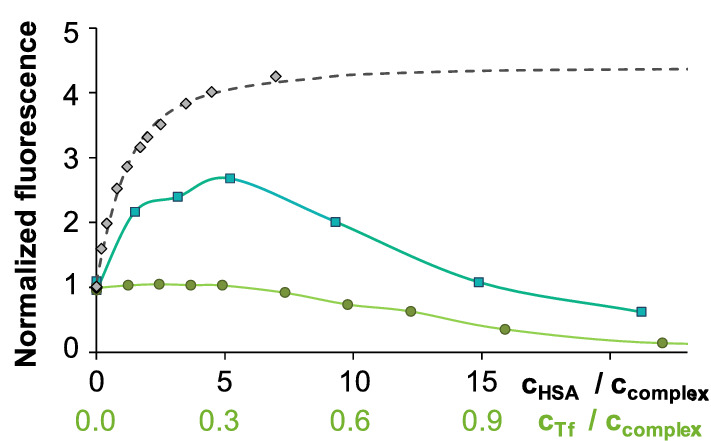
Changes of fluorescence intensity in the InQ_3_ – HSA – Tf (■), InQ_3_ – Tf (●) and InQ_3_ – HSA (♦,---) ternary systems normalized for the emission intensity of the complex at λ_EM_ = 550 nm. Plotted values are derived from Fig. 7 (ternary system), Figure S5 (Tf) and Fig. 3 and the computed binding constant (log*K*’ = 5.1) calculated for the InQ_3_ – HSA system. {*c*_InQ3_ = 10 μM; pH = 7.40; λ_EX_ = 367 nm; equilibration time: 6 h; spectra are corrected by self-absorbance and inner filter effect}

In these experiments, the applied HSA-to-Tf ratio corresponds to their physiological ratio in blood serum (630:38). Figure 8 depicts the intensity changes of the ternary system (complex and both proteins) for InQ_3_ at λ_EX_ = 367 nm and λ_EM_ = 530 nm in comparison to the respective binary systems (complex and only one type of protein). Apparently, albumin binding of the complex is indeed achieved in the ternary system, although to a lower extent compared to the InQ_3_ – HSA binary system, and the Tf binding of In(III) becomes more significant at higher protein concentrations. Consequently, the measured intensity reduces parallel with the decreasing (free and albumin bound) InQ_3_ concentration. As Fig. 7 shows, GaQ_3_ and InQ_3_ complexes behave similarly at smaller protein concentrations added, and the moderate emission intensity of both complexes (10 μM) is increased by the addition of a mixture of HSA (5.2 equiv.) and Tf (0.32 equiv.). Based on these spectra, InQ_3_ and GaQ_3_ are at least partly HSA-bound. However, minor dissociation of the complexes is evidenced by the UV–vis spectra (Fig. S7) even at this ratio that is more advanced for InQ_3_ than for GaQ_3_ (*ca.* 30% and 10% metal ion is Tf bound, respectively). The differences between the two complexes become more pronounced by getting closer to the physiologically relevant protein concentrations. In the presence of 212 μM HSA and 12.8 μM Tf (ratio: 1:21.2:1.28), the fluorescence of InQ_3_ is diminished, as most of the complex decomposes and In(III) is bound to Tf. On the other hand, the GaQ_3_ containing sample is still highly fluorescent revealing significant albumin binding of the non-dissociated complex. UV–vis spectra confirmed the more moderate (25%) dissociation of GaQ_3_ at this ratio compared to the practically completely dissociated InQ_3_ (Fig. S7). To mimic the blood protein concentrations, three-times higher values would be needed; however, under that conditions fluorometric detection becomes inadequate due to the too high absorbance and light scattering of the samples.

**Fig. 9:**
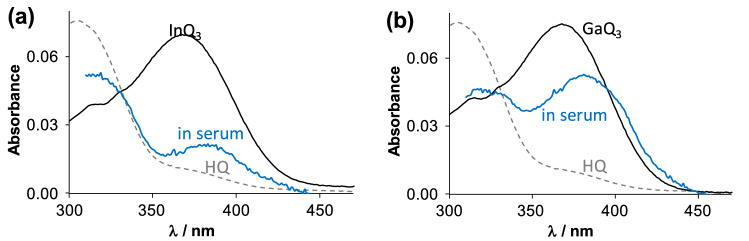
UV–vis absorbance spectra recorded for InQ_3_ (**a**) and GaQ_3_ (**b**) in the absence (black lines) and presence of threefold diluted blood serum (blue lines) plotted together with the spectrum of free HQ (grey dotted line). Absorbance spectra of the mixtures are subtracted by the spectrum of blood serum. {*c*_complexes_ = 10 μM; blood serum: filtered on 1.2 μm filter and threefold diluted with buffer; *ℓ* = 1 cm; pH = 7.40, equilibration time: 6 h}

With this knowledge, samples prepared in blood serum were studied by UV–vis spectrometry to count the significance of HMM and LMM serum components in the speciation of the complexes (Figs. 9 and S8). Unfortunately, fluorescence spectra could not be recorded due to the significant light scattering of the samples. Interestingly, the chemical equilibrium was reached within 10 min between 10 µM GaQ_3_ and threefold diluted serum based on the recorded UV–vis spectra, while 4 h was needed when InQ_3_ was studied under the same conditions (Fig. S8). UV–vis spectra in Fig. 9 resemble the behaviour observed for the complex – HSA – Tf ternary systems (Fig. S7) with some important differences. First, the λ_max_ of GaQ_3_ not only decreased slightly but also shifted to 382 nm in blood serum, while the absorption at 310 nm (that corresponds to the free ligand) did not increase in line with the decrease of the former band. This refers to the altered coordination sphere of the metal complex. With InQ_3_ the complex dissociation is more pronounced (see increasing absorption at 310 nm); at the same time, the highly reduced complex band also shifts to 385 nm. Keeping in mind the difficulties of interpretation of UV–vis spectroscopic data obtained in complex matrices, some assumptions can be made relying on the results of binary and ternary systems. Significant Tf binding of In(III) in blood serum is likely, while GaQ_3_ interacts less with this protein. The complex band was shifted only in serum samples; the presence of HQ – Fe(II)/(III) or Cu(II) complexes can be excluded on the basis of the obtained spectra [49, 50]. Formation of HQ – Mg(II) complex is not feasible even at physiological Mg(II) ion levels (0.7–1.0 mM) based on the available stability constants [51]. Zn(II) ion forms higher stability complexes with HQ as Mg(II) [51, 52] and could compete for HQ with In(III) or Ga(III) if its total blood plasma concentration (10–28 μM) would be freely available. Although Zn(II) ion forms complexes rapidly in general [53], the vast majority of this metal ion is bound to HMM (α-1 antitrypsin, HSA) and LMM serum components (histidine) in blood [54], therefore, the formation of Zn(II) – HQ complexes is not likely. The presence of mixed-ligand In(III)/Ga(III) – HQ complexes is assumed with *e.g.* amino acids or small peptides.

Noteworthy, In(III) – Tf binding constants are smaller than those of Ga(III), at the same time, the stronger hydrolysis of Ga(III) ions at pH 7.4 in comparison to In(III) ions leads to a reversal in apparent stability trend under the conditions applied. Our results suggest somewhat higher In(III)-Tf binding constants under the present conditions in comparison to the values reported by Harris et al*.* [18]. This way it is no surprise that complex InQ_3_ seems to be more prone to dissociate upon interaction with Tf (even in blood serum) in comparison to GaQ_3_.

## Conclusions

Solution speciation of In(III) complexes of four bidentate ligands (maltol, deferiprone, HQ and HQS) was studied in pure water in the presence of 0.20 M chloride ions. On the basis of the determined stability constants, *tris-*ligand complexes predominate at pH 7.4 in most cases and their stability follows the trend of HQ > deferiprone, HQS >  > maltol. Despite the higher overall stability constants of the *tris-*HQ complex formed with Ga(III) over In(III), the stronger hydrolysis tendency of Ga(III) ions results in a somewhat lower conditional stability at pH 7.4. Directly measured and calculated fractions of the *tris-*ligand complexes at 10 µM concentration and at pH 7.4 reveal very low stability for the *tris-*maltolato complex (< 20%), while non-dissociated InQ_3_ and InD_3_ are present in solution in *ca.* 90%. InQ_3_ displays reasonable stability (80% *tris*-ligand complex) in the presence of low molecular mass chelate forming ligands of blood plasma such as citrate, oxalate and phosphate.

Binding of InCl_3_ and the *tris-*ligand complexes InD_3_, InM_3_, InQ_3_ and its Ga(III) analogue GaQ_3_ to human serum albumin and human transferrin was monitored by steady-state and time-resolved spectrofluorometry, UV–vis spectrophotometry and membrane ultrafiltration. Our results revealed the moderate binding of InQ_3_ to HSA (log*K*′ = 5.0–5.1). This binding constant is one order of magnitude higher than that reported for the GaQ_3_ – HSA interaction. The fluorescence lifetimes of InQ_3_ and GaQ_3_ were determined and bi-exponential decays for the free and albumin-bound forms were found, respectively. The more hydrophilic InD_3_ can bind in a much lower extent to HSA. ApoTf can displace HQ, deferiprone and maltol effectively from their In(III) complexes. Substitution of deferiprone and maltol occurs to a much higher extent compared to that of HQ, while Tf binding of non-dissociated InQ_3_ was also observed at high complex-to-Tf ratios, which could not be detected for GaQ_3_. Studies conducted with the InQ_3_/GaQ_3_ – HSA – Tf ternary systems reveal the more pronounced Tf binding of In(III), while Ga(III) remains preferably in the GaQ_3_ complex and binds to HSA to a greater extent. In human blood serum, a very similar role of Tf is presumed, and the remaining complexes may interact partly with LMM serum constituents. This behavior explains why ^111^InQ_3_ cannot be administered into the blood directly and a previous blood cell labelling procedure is necessary, while circulating GaQ_3_, at least partly, can reach the target cells.

## Experimental

### Chemicals

InCl_3_, deferiprone, HQ, HQS, maltol, citric acid, oxalic acid, 4-(2-hydroxyethyl)-1-piperazineethanesulfonic acid (HEPES), HSA (product Nr.: *A8763*, essentially globulin free), apoTf (iron-free form, *T2036*), Tf containing physiological amount of iron (product Nr.: *T3309*, loaded with Fe(III) in 20–40%) and human serum (from male AB plasma, *H4522*) were purchased from Sigma-Aldrich. Inorganic chemicals such as KOH, KCl, HCl, NaCl, NaH_2_PO_4_, NaHCO_3_ were products of Molar Chemicals or Reanal.

### Stock solutions and sample preparation

For the preparation of stock solutions and samples, Milli-Q water was used. Exact concentration of ligand stock solutions was determined via pH-potentiometric titrations (vide infra). InCl_3_ and GaCl_3_ stock solutions were prepared by the dissolution of the anhydrous salts in a known amount of HCl, and the concentration was determined by complexometry via the EDTA complexes. Stock solutions of the *tris*-ligand In(III) and Ga(III) complexes were prepared by mixing the metal ion and the corresponding ligand in 1:3 ratio in water, *c*_complex_ = 30–200 μM. Protein stock solutions were prepared in 10 mM HEPES buffer containing 100 mM NaCl and 25 mM NaHCO_3_ (pH = 7.40) and their concentration was calculated on the basis of their UV–Vis absorbance: *ε*_280 nm_(HSA) = 36,850 M^−1^ cm^−1^, *ε*_278 nm_(apoTf) = 92,300 M^−1^ cm^−1^ [55, 56]. The concentration of Tf stock solutions was determined based on the average molar weight reported by the producer and calculated molar absorbance at 280 nm fall between the reported values of apoTf and holoTf [57]. Serum was filtered on 1.2 μm polyethersulfone syringe filters (OlimPeak, Teknokroma) and diluted with the buffer. The same buffer was used for sample preparation in protein binding studies. Samples containing the complexes InQ_3_, InD_3_ or InCl_3_ and apoTf or Tf were incubated for 6 h, 1 h and 3 h, respectively. All samples were incubated at room temperature.

### pH-potentiometry

The pH-potentiometric measurements for the determination of the proton dissociation constants of the ligands (HQS, maltol, deferiprone) and the overall stability constants of their In(III) complexes were carried out at 25.0 ± 0.1 °C in water at an ionic strength of 0.20 M (KCl, Sigma-Aldrich). The titrations were performed with a carbonate-free KOH solution of known concentration (0.20 M). An Orion 710A pH-meter equipped with a Metrohm combined electrode (type 6.0234.100) and a Metrohm 665 Dosimat burette were used for the pH-potentiometric measurements. The electrode system was calibrated to the pH =  − log[H^+^] scale using the method suggested by Irving et al*.* [58]. The average water ionization constant, pK_w_, is 13.76 ± 0.01, which corresponds well to literature data [59]. The reproducibility of the titration points included in the calculations was within 0.005 pH units. The pH-potentiometric titrations were performed in the pH range 2.0–11.5. The initial volume of the samples was 10.0 mL, and the ligand concentration was 3 mM and metal ion-to-ligand ratios of 1:1 to 1:4 were used. The fitting of the titration curves was less than 0.01 mL. (The fitting parameter is the average difference between the experimental and calculated titration curves expressed in the volume of the titrant.) Samples were deoxygenated by bubbling purified argon through them for *ca.* 10 min prior to the measurements. The proton dissociation constants of the ligands, the stoichiometry and the overall stability constants of the complexes were determined with the computer program HYPERQUAD [60]. Overall stability constants of the complexes (*β*(M_*p*_L_*q*_H_*r*_)) is defined for the general equilibrium *p*M + *q*L + *r*H $\rightleftharpoons$ M_*p*_L_*q*_H_*r*_ as *β* (M_*p*_L_*q*_H_*r*_) = [M_*p*_L_*q*_H_*r*_]/[M]^*p*^[L]^*q*^[H]^*r*^ where M denotes the metal ion and L the completely deprotonated ligand. Hydrolysis constants for In(III) and stability constants for the In(III)-chlorido complexes (Table 1) are taken from references [27, 28]. The calculations were always made from the experimental titration data measured in the absence of any precipitate in the solution.

### UV – visible spectrophotometry

An Agilent Cary 8454 diode array spectrophotometer was used to obtain UV–visible (UV–vis) spectra in the interval 190–1100 nm. The path length (*l*) was 1 or 2 cm.

Proton dissociation of the ligands (HQ, HQS, maltol, deferiprone) and overall stability constants and the individual spectra of their complexes were calculated with the computer program PSEQUAD [43]. The spectrophotometric titrations were performed on samples of the ligands with or without In(III); the concentration of the ligands was 0.10 mM, and the metal-to-ligand ratios were 1:1, 1:2, 1:3 and 1:4 over the pH range used in pH-potentiometric measurements at 25.0 ± 0.1 °C at an ionic strength of 0.20 M (KCl).

For (apo)Tf containing samples, complex concentrations were 20 μM and 0–2.5 equiv. protein was added. Reference spectra for the complexes, ligands and protein were recorded as well. Samples prepared with blood serum contained three-fold diluted serum and 10 μM metal complexes. UV–vis spectra presented in this paper for complexes InD_3_ or InQ_3_ are subtracted by the spectrum of reference protein (HSA (apo)Tf or serum) sample in all cases in favour of the better interpretation of the results.

### Spectrofluorometry

Fluorescence studies were implemented by a Fluoromax (Horiba Jobin Yvon) fluorometer in 1 cm quartz cells. Samples contained 1 μM HSA or (apo)Tf and various protein-to-metal complex (or InCl_3_) ratios (up to complex/HSA = 1:30) were used. The excitation wavelength was 295 nm and 280 nm for HSA and Tf binding studies, respectively; emission intensity was registered between 300 and 450 nm. Intrinsic fluorescence of complex InQ_3_ was also followed at *λ*_EX_ = 367 nm, and *λ*_EM_ = 450 – 650 nm. In this setup, the complex concentration was *ca.* 10 or 20 μM, and protein (HSA, (apo)Tf) concentration was varied between 0 and 130 μM, and residual fluorescence of the proteins were subtracted if it was necessary. Corrections for self-absorbance and inner filter effect were done as described in our former work using the formula suggested by Lakowicz [22, 44]. Computer program PSEQUAD [43] was utilized for the calculation of binding constants (*K*’) for HSA – InQ_3_ adducts similar to the approach described in our former works [22, 61]. Calculations were always based on data obtained from at least two independent measurements.

Three-dimensional spectra were recorded for ternary systems containing InQ_3_ or GaQ_3_, HSA and Tf in the wavelength range *λ*_EX_ = 250 – 450 nm and *λ*_EM_ = 300 – 700 nm. All three-dimensional spectra were corrected by residual fluorescence of the proteins, inner filter effect and self-absorbance.

Fluorescence lifetime was measured on the same fluorometer equipped with a DeltaHub time-correlated single photon counting (TCSPC) controller applying NanoLED light sources N-300 (λ_max_ = 300 nm) and N-350 (*λ*_max_ = 355 nm) (Horiba Jobin Yvon). Further instrumental settings are listed in Table S2. The background (obtained with blank samples) was subtracted from the decay of the samples. The program DAS6 (version 6.6.; Horiba, Jobin Yvon) was used for the analysis of the experimental fluorescence decays. The fluorescence intensity decay over time is described by a sum of exponentials,

1 $$I\left(t\right)={\sum }_{i=1}^{n}{\alpha }_{i}\mathrm{exp}\left(\frac{-t}{{\tau }_{i}}\right)$$

where *α*_*i*_ and *τ*_*i*_ are the normalized amplitude and lifetime of component *i* respectively [44]. The quality of the fit was judged from a χ^2^_R_ value close to 1.0 and a random distribution of weighted residuals.

### Ultrafiltration

Samples containing InD_3_ and apoTf were separated by ultrafiltration through 10 kDa membrane filters (Millipore, Amicon Ultra-0.5) into low and high molecular mass (LMM and HMM) fractions as described in our former works [22, 61]. Samples contained 20 μM complex and 0–33 μM apoTf. The concentration of the non-bound complex and ligand in the LMM fractions was determined by UV–vis spectrophotometry by comparing the recorded spectra to those of reference samples without the protein.

## Supplementary Information

Below is the link to the electronic supplementary material.

Supplementary file1 (PDF 2157 KB)

## Abbreviations

apoTf: Apotransferrin
deferiprone: 3-Hydroxy-1,2-dimethylpyridin-4(1*H*)-one
GaM_3_: *Tris*(3-Hydroxy-2-methyl-4*H*-pyran-4-onato)gallium(III)
GaQ_3_: *Tris*(8-Quinolinolato)gallium(III)
HEPES: 4-(2-Hydroxyethyl)-1-piperazineethanesulfonic acid
HMM: High molecular mass
HQ: 8-Hydroxyquinoline
HQS: 8-Hydroyquinoline-5-sulfonate
HSA: Human serum albumin
InD_3_: *Tris*(3-Hydroxy-2-methyl-4*H*-pyran-4-onato)indium(III)
InM_3_: *Tris*(3-Hydroxy-2-methyl-4*H*-pyran-4-onato)indium(III)
InQ_3_: *Tris*(8-Quinolinolato)indium(III)
LMM: Low molecular mass
maltol: 3-Hydroxy-2-methyl-4*H*-pyran-4-one
Tf: Transferrin
UV–vis: UV–visible
