# Supplementary material for: Solution speciation and human serum protein binding of indium(III) complexes of 8-hydroxyquinoline, deferiprone and maltol
Source: J Biol Inorg Chem. 2022 Mar 3;27(3):315–28. doi: 10.1007/s00775-022-01935-6 (PMC8960621; doi:10.1007/s00775-022-01935-6)
Supplement: Supplementary file 1 — Supplementary file1 (PDF 2157 KB) [file 775_2022_1935_MOESM1_ESM.pdf]

SUPPLEMENTARY INFORMATION

Solution speciation and human serum protein binding of indium(III) complexes of 8-hydroxyquinoline, deferiprone and maltol

Orsolya Dömötör, Bernhard K. Keppler, Éva A. Enyedy

FIGURES

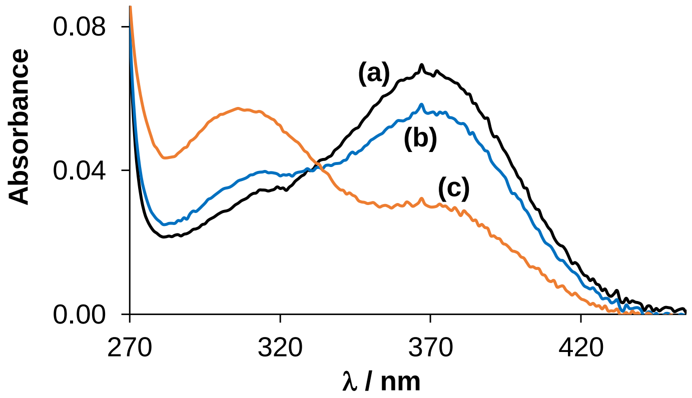

Fig. S1 UV-vis absorbance spectra of InQ_3 at pH 7.4 (a) in the absence and (b) in the presence of LMM component mixture of phosphate, citrate and oxalate applied in their serum concentration, and (c) in their tenfold serum concentration. {$c_{complex}$ = 10 µM; serum concentration of phosphate = 1.1 mM, citrate = 99 µM and oxalate = 9.3 µM; ℓ = 1 cm; equilibration time: 6 h}

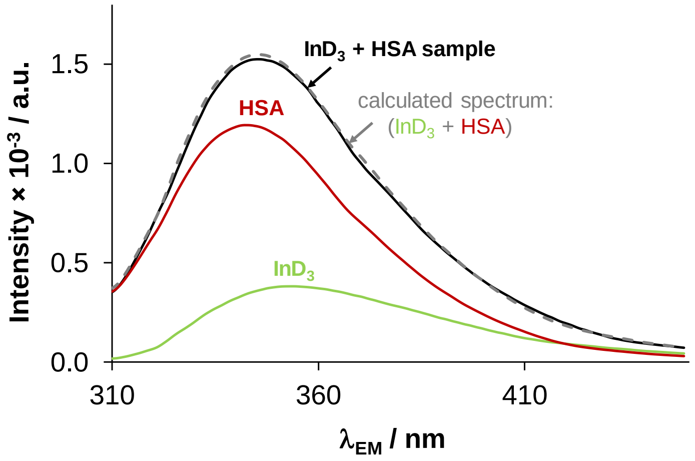

Fig. S2 Fluorescence spectra of InD_3 (green line), HSA (red line) and the mathematical sum of the two spectra (grey dashed line) and the measured spectrum of the InD_3 – HSA system (black solid line). {$c_{complex}$ = 7.6 μM; c_{HSA} = 1.0 μM; λ_{EX} = 295 nm; pH = 7.40}

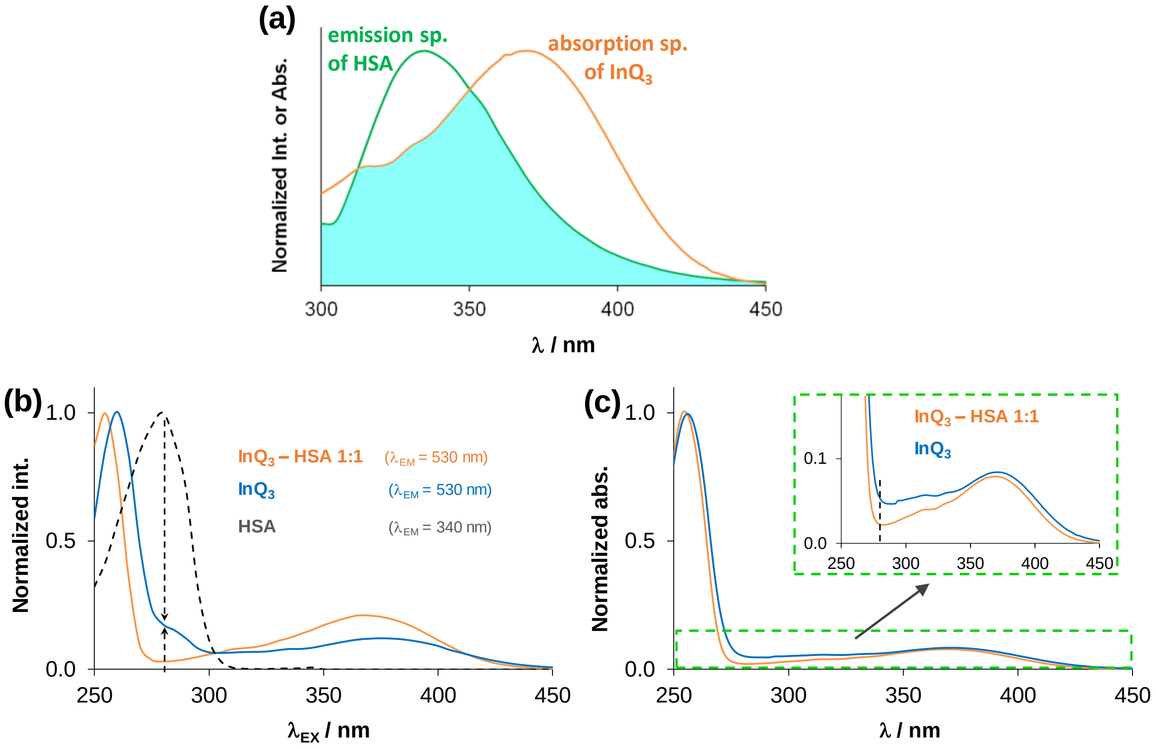

Fig. S3 (a) Spectral overlap between the fluorescence emission spectrum of HSA and UV-vis absorbance spectrum of InQ_3. (b) Normalized fluorescence excitation spectra, and (c) normalized absorbance spectra of the indicated systems. Absorbance of the InQ_3 – HSA sample is subtracted by the absorbance of HSA. {$c_{complex}$ = 10 μM; c_{HSA} = 10 μM; λ_{EX} = 295 nm (HSA); pH = 7.40}

FRET process occurs when the emission spectrum of a fluorophore (donor) overlaps with the absorption spectrum of another molecule (acceptor) and they are close enough to evolve a dipole–dipole interaction [44]. This spectral overlap is seen in Fig. S3a. The assumed mechanism is also supported by the appearance of a definite shoulder in the fluorescence excitation spectrum of InQ_3 at λ_{EX} = c.a. 280 nm in the presence of HSA, which does not appear in the absorbance spectrum of the same system, and it is neither characteristic for free InQ_3 itself (see Fig. S3b and c). λ_{EX} = 280 nm is the excitation maximum of HSA, namely the excitation of albumin results in fluorescence of the metal complex due to the energy transfer.

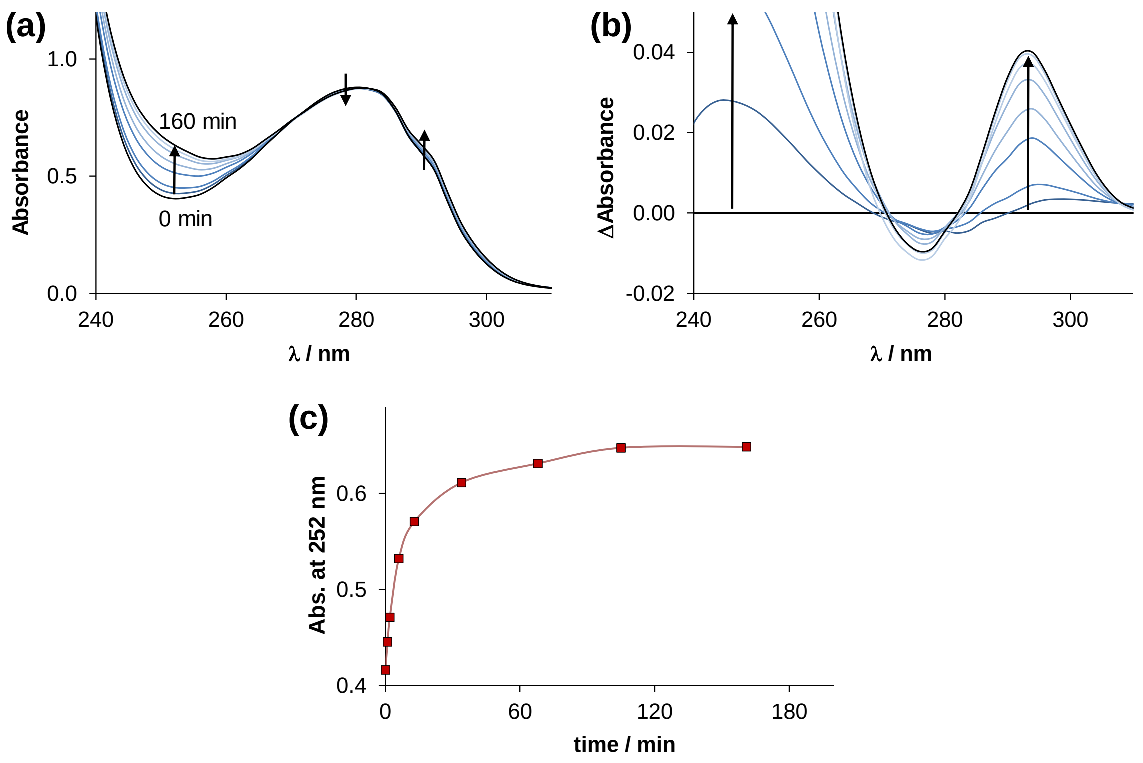

Fig. S4 (a) Time dependence of the UV-vis absorbance spectra of apoTf – $InCl_3$ 1:2 system, and (b) calculated difference spectra for the same system. (c) Absorbance changes at 252 nm are plotted as a function of time for apoTf – $InCl_3$ 1:2 (■) {c_{apoTf} = 11.5 μM; c_{InCl_3} = 20.8 μM; ℓ = 1 cm; pH = 7.40}

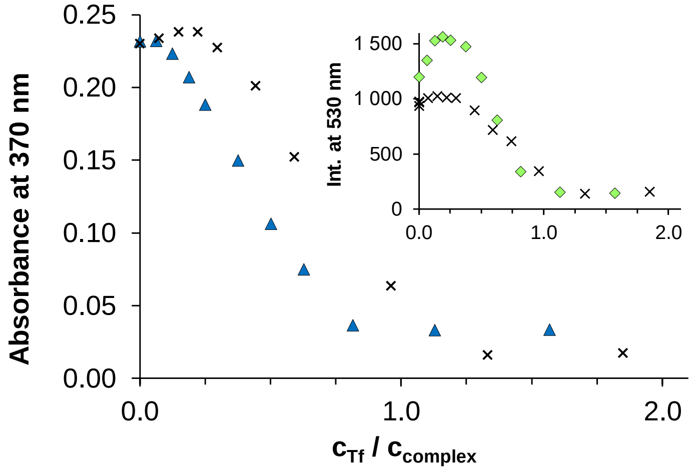

Fig. S5 UV-vis absorbance values of InQ_3 at 370 nm in the absence and presence of various amounts of apoTf (▲) or Fe-Tf (×). Inset shows the fluorescence intensities recorded for the same systems: InQ_3 – apoTf (♦) and InQ_3 – Fe-Tf (×). Absorbance spectra depicted here are subtracted by the spectrum of apoTf. {$c_{complex}$ = 19 μM; c_{apoTf} = 0 – 30 μM; ℓ = 2 cm; inset: λ_{EX} = 367 nm, ℓ =1×1 cm; pH = 7.40, equilibration time: 6 h}

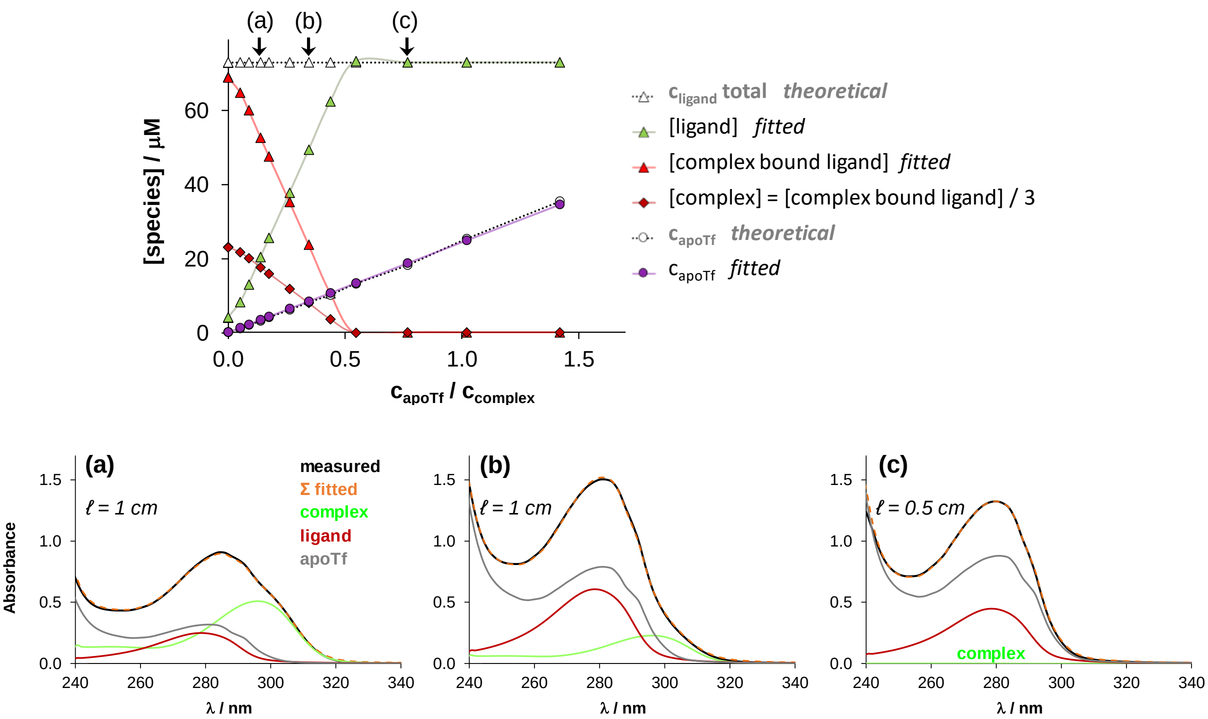

Fig. S6 Concentration distribution curves calculated on the basis of spectral deconvolution of the UV-vis spectra recorded for the InD_3 – apoTf system, where 22 µM complex was 'titrated' by 0 – 31 µM apoTf. Figures a-c show the measured, and deconvoluted UV-vis spectra at the apoTf-to-complex ratios of 0.13 (a), 0.34 (b) and 0.77 (c). {ℓ = 1 cm (a,b) or 0.5 cm (c); pH = 7.40}

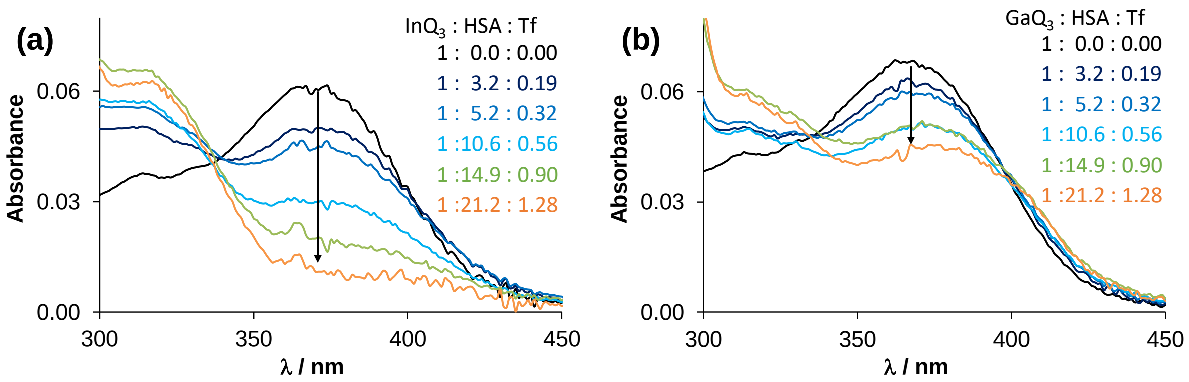

Fig. S7 (a) UV-vis absorbance spectra recorded for InQ_3 – HSA – Tf and (b) GaQ_3 – HSA – Tf systems at the indicated compositions. The HSA-to-Tf ratio corresponds to the physiological ratio of the two proteins in blood serum. Tf contains iron(III) in physiological amount. Absorbance spectra depicted here are subtracted by the spectrum of the corresponding protein mixture. {$c_{InQ3} = c_{GaQ3}$ = 10 µM, c_{HSA} = 0 – 212 µM; c_{Tf} = 0 – 12.8 µM; pH = 7.40, equilibration time: 6 h}

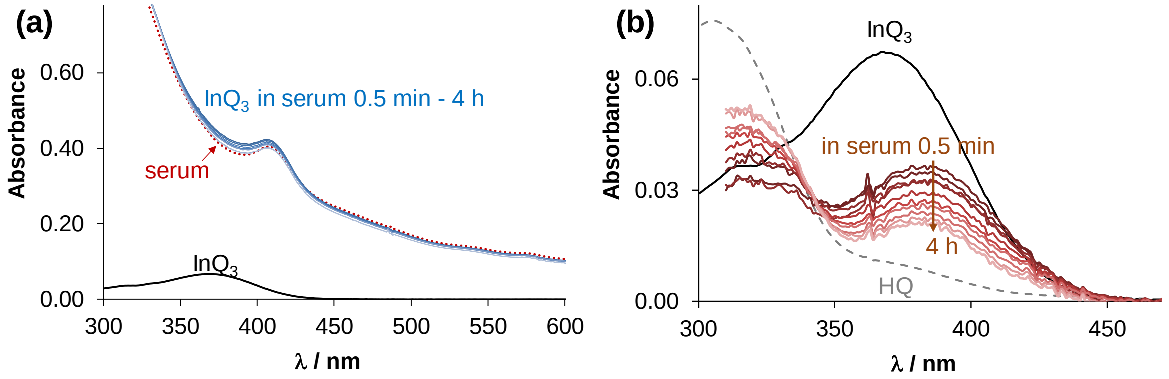

Fig. S8 Primary time dependent UV–vis absorbance spectra of the InQ_3 – diluted serum system (a) and the spectra subtracted by the absorbance of serum (b) plotted together with the spectra of InQ_3 (black line) and HQ (grey dashed line). {$c_{complex}$ = 10 μM; c_{HQ} = 30 μM; serum: filtered on 1.2 μm filter and 3-fold diluted with buffer; ℓ = 1 cm; pH = 7.40, equilibration time: 6 h}

TABLES

Table S1. Calculated pM values for the studied In(III) complexes and their Ga analogues, where pM = –log[M(III)].

As both metal ions form hydroxido complexes at pH 7.4 (chlorido complex formation of In(III) is negligible at this pH), the use of pIn and pGa values (instead of pIn and pGa*) is not correct, since they do not account the hydrolysis of the metal ions.*

**pM**	**deferiprone**	**maltol**	**HQS**	**HQ**
pIn	16.25	14.31	16.23	16.48
pGa	21.59[a]	18.76[b]	20.67[b]	20.61[b]

[a] Computed on the basis of stability constants taken from Ref. [27]. [b] Computed on the basis of stability constants taken from Ref. [29]

Table S2. Instrument parameters for TCSPC measurements

**Fluorophore**	**InQ_3 or GaQ_3**	**InD_3**
LED source λ_{EX} (nm)	N-350	N-300
λ_{EM} (nm)	535	360
Slit width on the emission side (nm)	7-10	15
Count Nr. at peak channel	10 000	
Approx. Nr. of channels used for analysis *	2000	400
Time window	100 ns	
Time calibration (ns/ch)	0.02532	
Instrument response function	Ludox®	

* total number of channels = 4096
